# Non-random fragmentation patterns in circulating cell-free DNA reflect epigenetic regulation

**DOI:** 10.1186/1471-2164-16-S13-S1

**Published:** 2015-12-16

**Authors:** Maxim Ivanov, Ancha Baranova, Timothy Butler, Paul Spellman, Vladislav Mileyko

**Affiliations:** 1 Institute of Chemical Biology and Fundamental Medicine, Siberian Branch of the Russian Academy of Sciences, Prosp. Lavrentieva, 8, 630090, Novosibirsk, Russia; 2 Atlas Oncology Diagnostics, ltd, Moscow, 121069, Russia; 3 Moscow Institute of Physics and Technology, Dolgoprudny, Moscow Region, 141700 Russia; 4 Research Centre for Medical Genetics, Moscow, 115478, Russia; 5 School of Systems Biology, George Mason University, Fairfax, VA, 22030, USA; 6 Knight Cancer Institute, Oregon Health & Sciences University, Portland, OR 97239, USA; 7 Department of Molecular and Medical Genetics, Oregon Health & Sciences University, Portland, OR 97239, USA

## Abstract

**Background:**

The assessment of cell-free circulating DNA fragments, also known as a "liquid biopsy" of the patient's plasma, is an important source for the discovery and subsequent non-invasive monitoring of cancer and other pathological conditions. Although the nucleosome-guided fragmentation patterns of cell-free DNA (cfDNA) have not yet been studied in detail, non-random representation of cfDNA sequencies may reflect chromatin features in the tissue of origin at gene-regulation level.

**Results:**

In this study, we investigated the association between epigenetic landscapes of human tissues evident in the patterns of cfDNA in plasma by deep sequencing of human cfDNA samples. We have demonstrated that baseline characteristics of cfDNA fragmentation pattern are in concordance with the ones corresponding to cell lines-derived. To identify the loci differentially represented in cfDNA fragment, we mapped the transcription start sites within the sequenced cfDNA fragments and tested for association of these genomic coordinates with the relative strength and the patterns of gene expressions. Preselected sets of house-keeping and tissue specific genes were used as models for actively expressed and silenced genes. Developed measure of gene regulation was able to differentiate these two sets based on sequencing coverage near gene transcription start site.

**Conclusion:**

Experimental outcomes suggest that cfDNA retains characteristics previously noted in genome-wide analysis of chromatin structure, in particular, in MNase-seq assays. Thus far the analysis of the DNA fragmentation pattern may aid further developing of cfDNA based biomarkers for a variety of human conditions.

## Introduction

The most basic structural unit of the chromatin is a nucleosome that is formed by the binding of DNA to histone octamers containing two monomers for each of the four core histones [1]. Within the nucleosome, the DNA encircles the protein core 1.7 times as a coil of approximate 147 base pairs (b.p.) in length [2]. On the DNA strand, the nucleosomes are separated from each other by the "linker" stretches of nucleotides, which can be up to about 80 b.p. long [3].

The nucleosomes play an important role in epigenetic regulation of gene expression programs by competing for binding with transcription factors or by interfering with RNA polymerase positioning and movement [4-7]. A number of studies performed in various model organisms and human cell lines have demostrated that the positioning of the nucleosomes on DNA is somewhat variable, and that they tend to relocate in tissue-specific positions that resemble gene expression programs executed in particular types of cells [8-12]. One of the rules of nucleosome positioning is the nucleosome depletion that accompanies transcription start sites (TSSs) of actively expression genes. Typically, the nucleosome depleted regions (NDRs) are located approximately 50 b.p. upstream of active TSSs and correspond to the displacement of the so-called strictly positioned nucleosome at the "-1" upstream site and the subsequent nucleosome at +1 position downstream of TSS in question, with gradual decresing stringency of nucleosomal location on both ends of TSS. The integrity of nucleosomal organization around TSS is essential for the maintenance of the correct gene expression pattern in a given cell. In particluar, this organization provides a fast and reliable way to recruit transcription complexes for genes that have to steadily produce large amounts of their mRNAs, whereas "weak" or "fuzzy" positioned nucleosomes with larger footprints are assciated with higher plasticity of gene expression that allows for rapid changes in mRNAs levels in response to a specific demand [13].

In healthy patients, cfDNA fractions are mostly derived from apoptosis of various normal cells that generate small fragments of cell-free DNA, whereas the cell-free circulating DNA of cancer patients represents a mix of apotosis, necrosis, autophagy, or mitotic catastrophe [14]. Necrosis produces relatively long fragments of DNA, about 10,000 b.p. in length, while in apoptosis, the activation of endogenous endonucleases lead to the cleavage of chromatin DNA into internucleosomal fragments [15]. This effect is commonly used for the detection of apoptosis in the DNA laddering and TUNEL assays. In the majority of somatic tissues, apoptotic cleavage of DNA results in the formation of fragments roughly 195 b.p. in length and multiples thereof, whereas the fragmentation pattern of the neuronal chromatin is characterized by size of ~165 b.p. As the repeatable length corresponds to single nucleosome size (with degraded DNA linkers), one may expect that the patterns of DNA degradation are guided by nucleosome positioning. Within the nucleosomal core, DNA is protected from nucleases by histones, whereas the linker is vulnerable to digestion, hence, variation in fragment size is explained by variations in linker length. Indeed, back in 1973, Hewish & Burgoyne demonstrated that treatment with endonuclease disrupts the bead-like structures of undigested chromatin in an ordered fashion and produces a typical "laddered" electrophoregram instead of a smear [16-18].

Nucleosome guided patterns of apoptotic DNA fragmentation may have important implications for the analysis of circulating nucleic acids. First, the cfDNA fragment copy number may depend on the nucleosomal positioning at given DNA locus. Therefore, PCR primer systems may need be tuned to the regions that would produce a higher level of DNA amplification. Second, the prevalence of certain DNA fragments may directly reflect nucleosome positioning within certain loci and, therefore, serve as a proxy for gene expression levels. One could imagine cfDNA based quantitative PCR systems that employs nucleosome positioning to approximate expression levels for certain pathogenetically important genes, thus, opening a novel field in biomarker research that we may tentatively call "fragmentomics". Unfortunately, no nucleosome fragmentation pattern studies are so far being focused on cfDNA, so this avenue for cfDNA-based fragmentomics remains unexplored. In this paper, we employ high throughtput sequencing of human cfDNA to analyze the properties of cfDNA fragmentation patterns.

## Methods

### Data processing

**Figure 1:**
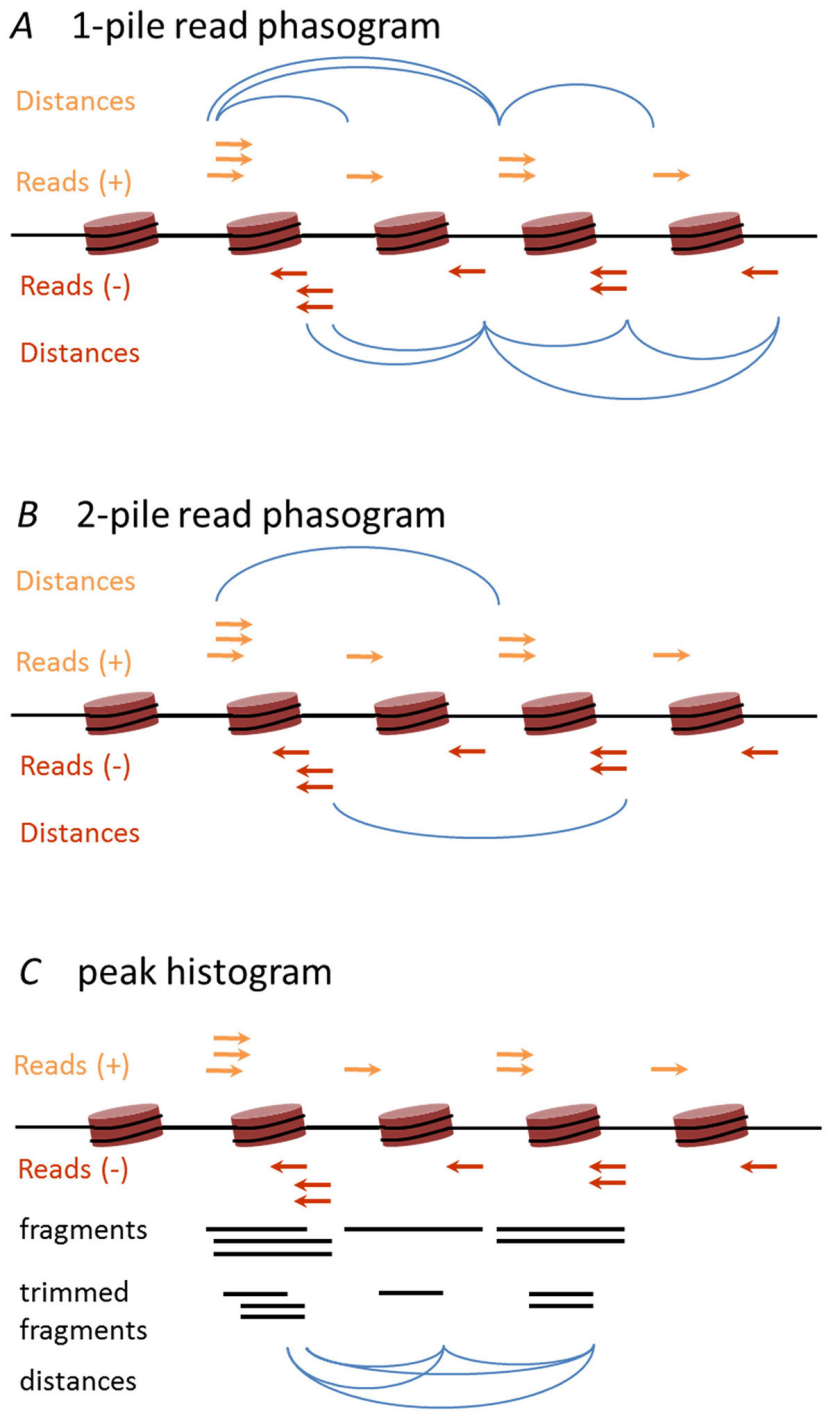
**Read phasogram and peak histograms calculating process**. *A*. Schematic description of the 1-pile read phasogram calculation. Blue arcs represent recorded distances between reads that map on the same strand. Not all distances are represented for better graphical visualization *B*. Schematic description of the 2-pile read phasogram calculation. Only distances between reads with at least 1 co-located read are taken into account. All distances taken into account are noted with blue arcs *C*. Schematic description of the peak histogram calculation. Paired end reads are coupled into fragments which are further trimmed by 40 b.p. around dyads so they all have length of 80 b.p. For the histogram calculation distances between trimmed fragments are recorded.

This study was performed on raw sequencing data published by Butler et. al. in their 2015 work of non-invasively sequencing of tumor genome [19]. The dataset we used consists of two samples of DNA from two patients: cell-free DNA from plasma of a patient with breast cancer (cfDNA sample 1 or cfDNA1) with paired nuclear DNA from leukocytes (genomic or leukocyte DNA) and cell-free DNA from plasma of a patient with sarcoma (cfDNA sample 2 or cfDNA2) without paired nuclear DNA. Both patients had progressive cancer with multiple metastases. Details on DNA extraction, purification and library preparation are provided by Butler et al. Of note, hybrid capture was conducted using Agilent SureSelectXT Human All Exon V4+UTRs kit. This brought some limitations for downstream analysis, which are mentioned in the text. Also it is important to note, that only leukocyte genomic DNA underwent sonication, while cell-free DNA sample libraries were sequenced without DNA fragmentation, which makes it possible to analyse cell-free DNA fragment distribution. All three libraries underwent 101b.p. sequencing on Illumina HiSeq 2000 instrument. Please refer to paper published by Butler et al. for the in depth information on patients enrollment, patients clinical history, experimental protocols and basic bioinformatics analysis of raw sequencing data. Based on SAM files all samples were anonymized before downstream analysis so only information about reads mapping positions was used. Unpaired reads and fragments with insert size of more then 1000 base pairs were removed. Read pairs were coupled further and resulting fragments were trimmed by 40 b.p. around dyads. For each sample, coverage function was built for each basepair position. Nucleosome position stringencies were calculated essentially as described in Valouev et al, using the software that performs the nucleosome mapping based on the kernel smoothed reads count calculation [20]. These nucleosome position stringencies are defined as genome regions between -73 and +73 b.p. positions centered around the mid-point at each nucleosome dyad and would be further refereed as peaks. In samples of cfDNA, 43% and 41% of exome were occupied by nucleosomes, while in leukocyte genomic DNA control, the nucleosome coverage was at 39%. Nucleosome peak calling was performed only for the limited genome regions distinctive by the long (>1000 b.p.) target sequence length. Cumulative length of these regions is 750000 b.p. 2193, 2095 and 1989 peaks were called for the 1st patient cfDNA data, 2nd patient cfDNA data and 1st patient nuclear DNA data. Genome coverage by nucleosomes was measured as the ratio between the cumulative called peak length and the genome length. Mononucleosome read phasograms were obtained as histogram of distances between codirectional reads (Figure 1). Number of piles indicated that only reads which have another N-1 or higher co-located reads were taken into account. For the analysis only 3-pile read phasograms were used. For the building of mononucleosome read phasograms, reads forming the minor fraction of fragments (with fragment length from 250 to 350) were ignored. Peak histograms are histograms of distance between called nucleosome peaks. For the building of mononucleosome and dinucleosome peak histograms different set of reads were used for the peak calling (which forms the major and the minor fraction of fragments respectively). Read phasograms were calculated based on the whole genome, wheares peak histograms were calculated based on the genome part, nucleosome calling of which was performed.

## Results and Discussion

### Overall distribution of cfDNA fragment lenght reflects apoptotic fragmentation

cfDNA samples of two female patients were paired-end sequenced without DNA sonication using Illumina high throuput technology. Additionally, in one of the patients, a sample of leukocyte genomic DNA was sequenced after sonication to serve as a control. To maximize coverage, whole exome plus UTR sequencing was performed instead of whole genome sequencing. For two cfDNA samples and the control DNA, 286 mln, 591 mln and 182 mln reads were obtained, respectively, an equivalent of 260×-840× coverage for each target region. Average read length was 100 b.p.

**Figure 2:**
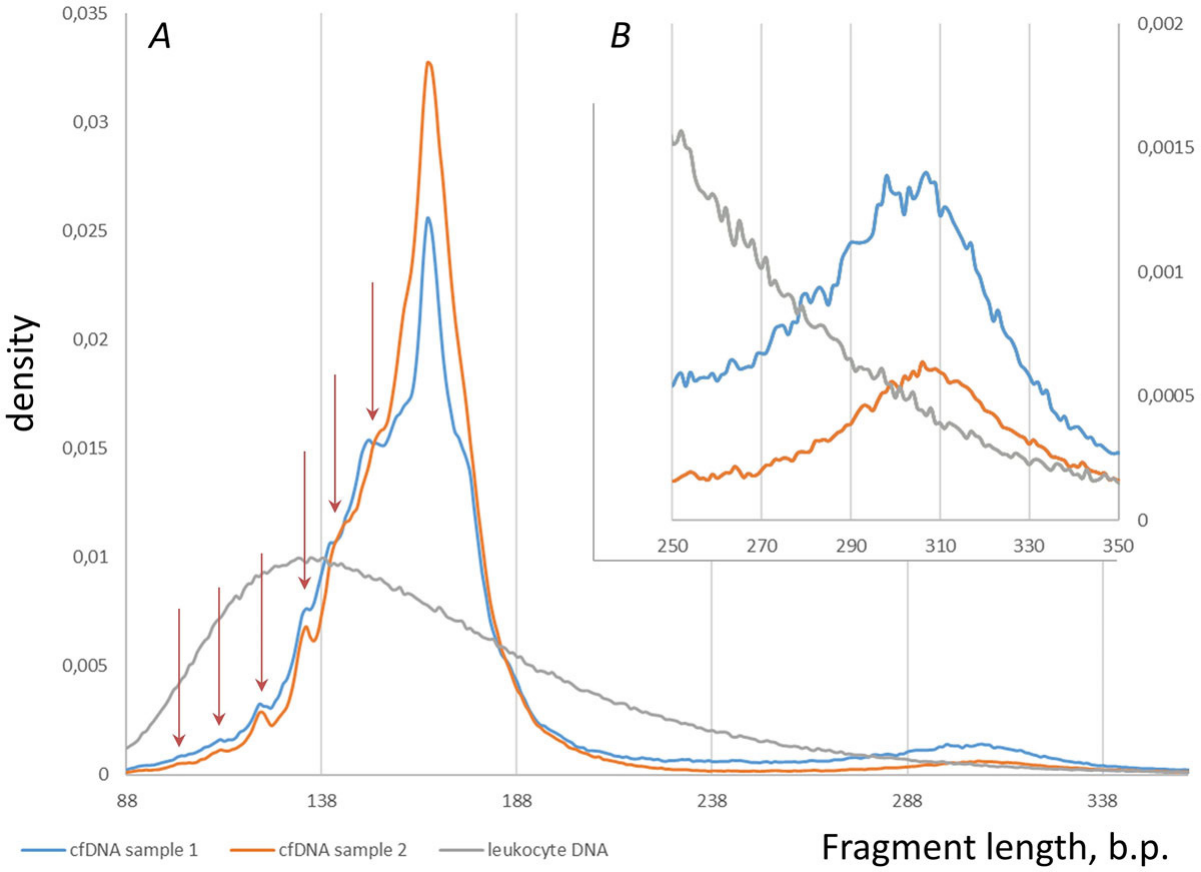
**Fragment length distributions**. For both cfDNA samples, the average fragment length was at 165 b.p., which corresponds to a single nucleosome. The dinucleosomal peak with average fragment length of 308 b.p. is also notable for both cfDNA samples. Panel *B *demonstrates zoom of panel *A*, representing only minor fraction of fragments. Red arrows note periodicity below major distribution peak.

After the coupling of paired cfDNA reads, the fragment length distribution graph was built (Figure 2A). This graph indicates that a major fraction of sequenced fragments has a mean length of 165 b.p. that roughtly correspond to the size of mononucleosome comprising to nucleosome core, H1 histone and some linker DNA, while a minor fraction of the fragments, with a mean length of 308 b.p., corresponds to dinucleosomes. Thus, overall distribution of the cfDNA fragments reflects apoptotic fragmentation. In the control DNA extracted from leukocytes, the distribution of the fragments sizes fitted the classic log-normal shape with the mode of 116 b.p. and average size of the fragments at 166 b.p.

Of note, the major cell-free DNA fraction demonstrates minor peaks at roughly 152, 143, 133, 122, 112 and 102 b.p. This effect of periodicity below the major peak has already been seen in fetal DNA [21], though periodicity pattern differs: peak at 152 b.p. was absent, the major peak was split on three signals and periodicity above major peak have been seen as well as it has been seen for longer reads (dinucleosome fraction of reads). Though the last three points may be explained by inconsistent coverage. Such 10 b.p. periodicity is similar to the pattern of nuclease cleavage of nucleosome-bound plasma DNA fragments and indicates that DNA molecules may be released from normal cells [22,23].

### In cfDNA, the depth of coverage reflects nucleosome positions

**Figure 3:**
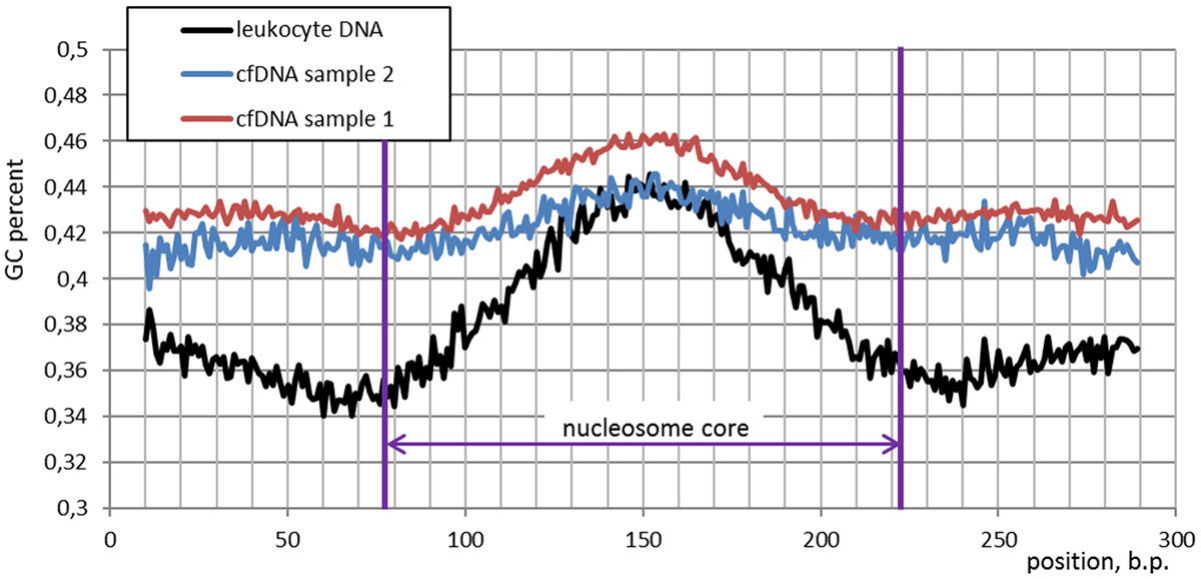
**Average GC content within called peaks (per each nucleotide position)**. As one can see, in leukocyte DNA peaks, the bias of GC content toward the peak center was substantially more pronounced.

On a typical fragment coverage track, a wave-like coverage depths pattern is observed, and is commonly explained by variations in GC content that affect efficiency of PCR during library preparation [24,25]. Moreover, employing hybridization as target DNA enrichment method during library preparation additionally increases bias towards coverage excess of GC-rich motifs. One could expect that these factors may significantly contribute to coverage function and process of nucleosome peak calling. This is confirmed by the fact that after nucleosome peak calling 39% of leukocyte genomic control DNA is occupied by peaks though nucleosome nature can not underlie them. In order to assess the degree of contribution of GC content to wave-like patterns in coverage an average GC content were calculated in both genomic and cfDNA, for each called peak (Figure 3). The mean GC contents of the cfDNA peak sequences (43.5 ± 12.1% and 42.5 ± 13.0% for the first and second cfDNA sample, respectively) significantly differs from the peaks in the genomic DNA (38.1 ± 11.9%), p < 0.0001. In genomic peaks, the bias of GC content toward the peak center was substantially more pronounced. This implies that for genomic DNA, the peaks are defined by their higher GC content to substantially larger degree that the peaks observed in cfDNA.

**Figure 4:**
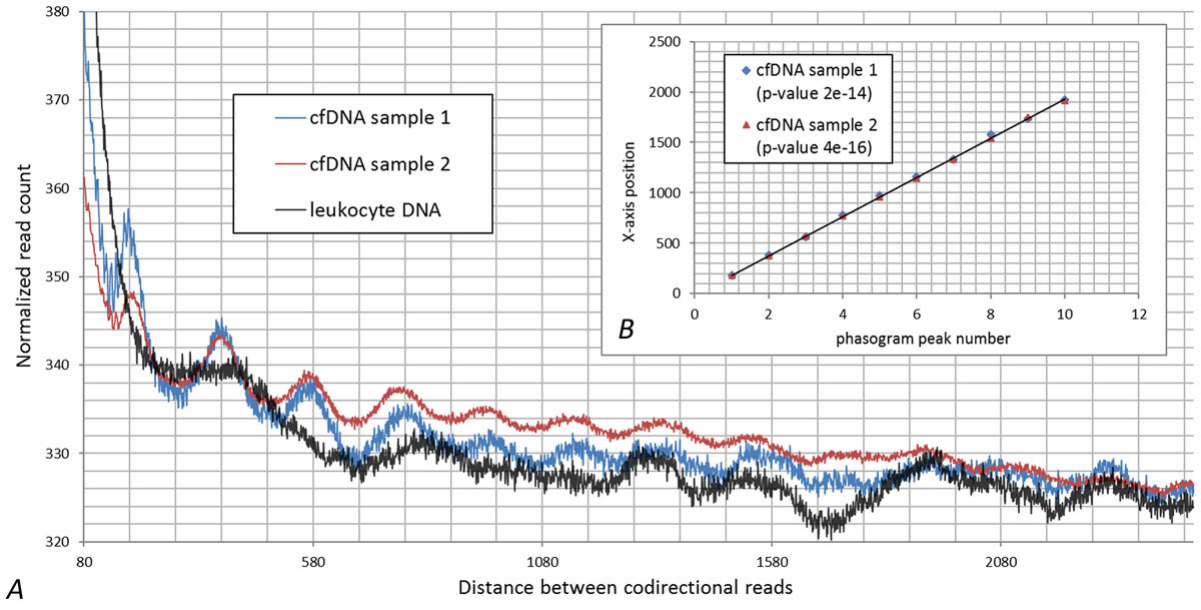
**Mononucleosome read phasogram**. Read phasogram is defined as histogram of distances between reads mapped to the same strand of human genome and demonstrated on panel *A*. For the calculation of mononucleosome read phasogram only reads that are coupled into fragments with length less than 250 are taken into account. As one can see, the mononucleosome read phasograms built for two different cfDNA samples correlate with each other while this pattern is not observed in leukocyte DNA sample. Panel *B *demonstrates the correlation of distance between peaks.

In order to describe the nucleosomal origin of cfDNA peaks, histograms of the distances between the reads mapped to the same strand of human genome or read phasograms were built (Figure 4). As one can see on the inset to Figure 4, the mononucleosome read phasograms built for two different cfDNA samples highly correlate with each other (Pearson' correlation coefficient equals to 1 up to the forth decimal place with p-value 1.2e-14), indicating the robustness of the technique. Moreover, for both libraries, the same spacing between the reads was observed (193 b.p.), which was comprised of the core size of 147 b.p. and a linker size of 46 b.p. These data are in concordance with a previous study of nucleosome occupancy in human cell lines (193-203 b.p. according to Valuoev et.al., 2011). The nucleosome-guided periodicity observed in cfDNA libraries is contrasted with a lack of periodicity observed in control library read phasogram. Therefore, the wave-like pattern in coverage depths of cfDNA depends on the nucleosome occupancy rather than biases introduced during the library amplification step. Nevertheless, GC content contribute to wave-like pattern of cfDNA data and may as pronounce single peaks as tail it of or even bring false positive calls. These results demonstrate the need of comprising the GC content during bioinformatics analysis of MNase-seq.

### Dinucleosome fragmentation pattern characterisation

**Figure 5:**
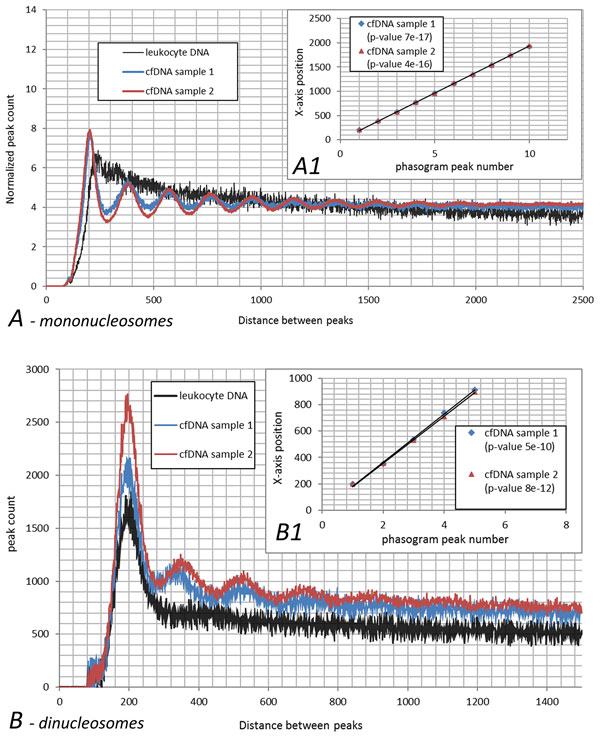
**Mononucleosome (*A*) and dinucleosome (*B*) peak histograms**. *A1 *and *B1 *inserts demonstrates correlation of distance between peaks. Peak histogram is histogram of distances between peak. For the building of mononucleosome (dinucleosome) peak histograms only reads that are coupled into fragments with length of less than 250 (with length of higher 250 and lower 350) are taken into account for peak calling.

For the analysis of the minor fraction of the fragments observed in cfDNA samples, the dinucleosome fragments, was done separately. If the dinucleosome processing is the same as for mononucleosomes, and represent mere underdigestion of DNA by endonucleases, one can expect that the histograms of distances between peaks (or peak histograms) of dinucleosome fragments observed in 2 different cfDNA samples would be similar. However, the dinucleosome peak histograms built for two cfDNA libraries demonstrate distinctly different patterns that were not the same as for mononucleosome peak histograms (Figure 5). In contrast to the mononucleosomal peak histogram built for genomic sample, the dinucleosomal one reveals a pronounced peak which is in accordance with the first peak in two cfDNA graphs. Moreover, for two cfDNA samples, dinucleosome peak histograms were discordant, with peak spacings being 182 b.p. in cfDNA1 and 174 b.p. in cfDNA2 (p < 0.01), while mononucleosomal peak histograms were concordant, with peak spacings at 192 b.p. and 193 b.p., respectively. Obviously, dinucleosome fragmentation patterns differ from that of the mononucleosomal ones. Further studies focusing at dinucleosome fragmentation pattern are necessary to understand whether this minor read fraction represents an interesting or useful cache for biomarker discovery.

### cfDNA fragmentation patterns correlate with known epigenetic marks

Chromatin remodelling is one of the major factors contributing epigenetic regulation [26]. In the mean time nucleosome organization is closely related to epigenetic marks, such as histone modifications and DNA methylation. Hence, in order to further assess the biological interpretation of the coverage function peaks, the association of fragmentation pattern and epigenetic marks was studied. For this purpose, H3k36me3, H3k4me2, H3k4me3, H3k09me3, H3k27ac, H3k27me3, H3k4me1, H3k79me2, H3k9ac, H4k20me1, Ezh2, H2az and Pol2b maps were downloaded from The Encyclopedia of DNA Elements (ENCODE) [27]. These maps show epigenetic marks in normal umbilical vein endothelial cells HUVEC, leukemic cell line K562 and normal epidermal keratinocytes Nhek. The intersection of the cfDNA mononucleosomal peaks with downloaded epigenetic marks mapped in three studied ENCODE datasets was performed.

Of note, cfDNA is highly heterogeneous since it represents numerous different tissues each of which has its own gene expression profiles. On the other hand, epigenetic regulation marks are basically tissue specific, the exact mechanism in which they marks contributes to the cfDNA fragment distribution is still unknown. Apparently, this is the main reason why we have seen no marks with a statistically signicant correlation with fragmentation patterns in cfDNA or why its also seen in genomic control. Though, in contrast to randomly selected sites in the targeted regions, the coverage peaks in both cfDNA samples were significantly (p < 0.01) associated with the RNA Polymerase II (Pol2b) signal - marker of actively transcribed chromatin - while in the nuclear DNA dataset, this association was not detected (p > 0.07). (Table 1). This demonstrates that chromatin changes associated with loci overall expression level contribute to the cfDNA fragmentation pattern.

**Table 1:** Association of epigenetic marks and DNA fragmentation patterns (statistical significance)

	K562			Huvek			Nhek		
	
	cfDNA1	cfDNA2	leukocyte DNA	cfDNA1	cfDNA2	leukocyte DNA	cfDNA1	cfDNA2	leukocyte DNA
H3k36me3	**+**	**+**		**+**	**+**	**+**	**+**	**+**	**+**

H3k4me2		**+**	**+**						

H3k4me3	**+**	**+**	**+**						

Pol2b	**+**	**+**		**+**	**+**		**+**	**+**	

Ezh2				**+**					

H2az					**+**		**+**	**+**	

H3k09me3				**+**	**+**	**+**	**+**	**+**	**+**

H3k27ac		**+**							**+**

H3k27me3	**+**		**+**		**+**			**+**	

H3k4me1	**+**	**+**	**+**	**+**	**+**				**+**

H3k79me2	**+**	**+**		**+**	**+**	**+**	**+**	**+**	**+**

H3k9ac		**+**						**+**	

H4k20me1	**+**			**+**	**+**		**+**	**+**	

Association of epigenetic marks and nucleosome fragmentation pattern in cfDNA (1 and 2 for the 1^st ^and 2d patient respectively) and nuclear DNA from leukocytes used as control. Following the peak calling for cfDNA and nuclear DNA comparison with epigenetic marks peaks obtained from ENCODE project was performed and p-values were calculated. Threshold of 0.05 was used to define statistically significant correlations, which were pointed by '+' in the respective cell. Cells respective to the statistically non-significant correlations were left empty.

### Association between gene expression and nucleosome fragmentation patterns

Associations between expression and nucleosome occupancy have been explored in the past several years in numerous studies. In a variety of cell lines, active gene promoters were shown to be nucleosome depleted. In this work, we tried to examine whether this trend reflects on cfDNA fragmentation patterns, or not.

In studied cfDNA samples, the capture targeted both the exome and UTR. Consequently, the regions immediately upstream of TSS could not be evaluated, and the significance of the most actively studied nucleosome-free region immediately upstream of the first TSS can not be evaluated. Additionally, the number of genes with the first exon that was large enough to study the nucleosome occupancy pattern was relatively small. Only 870 genes could be selected as having at least 700 nucleotides within first exon covered with probes starting from TSS, thus, enabling detection of the first 2.5 nucleosomes.

**Figure 6:**
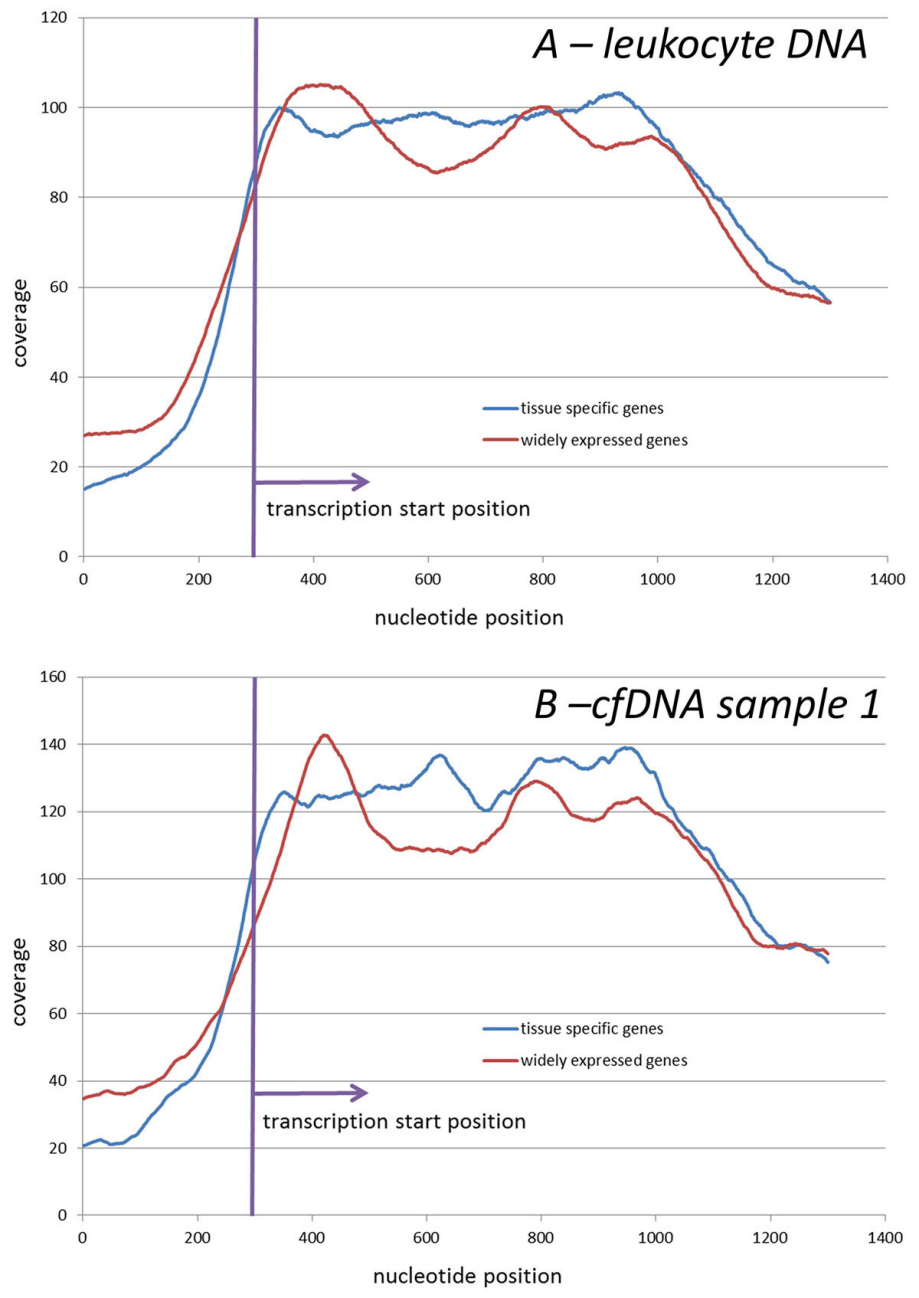
**Average per nucleotide coverage of gene around TSS for 134 tissue specific genes and 246 tissue non-specific genes for *A *leukocyte DNA and *B *paired cfDNA**. Target enrichment probes cover at least first 700 nucleotides of each selected gene downstream TSS.

As a model of actively expressed and silenced genes, tissue specific and housekeeping gene sets were employed. Tissue specific genes are silenced in the majority of human tissues and, therefore, the majority of cfDNA fragments corresponding for these genes will reflect the silenced gene pattern, while the housekeeping genes would be reperesented by the majority of the fragments coming form tissues where the gene is expressed. Among 870 genes with the longest first exons, 134 tissue specific (excluding those that are highly expressed in blood) and 246 widely expressed genes were selected using TiGER database. For each of these genes, average per nucleotide coverage of the region downstream of the TSS was plotted in cfDNA data and in the genome DNA extracted from leukocytes. (Figure 6). As can be seen, in both datasets per nucleotide coverage downstream TSS reflects the classic silenced and highly expressed gene patterns. In contrast to widely expressed housekeeping genes, tissue specific genes correspond to well-resolved +1, +2 and +3 peaks that are detected with decreasing stringency.

**Figure 7:**
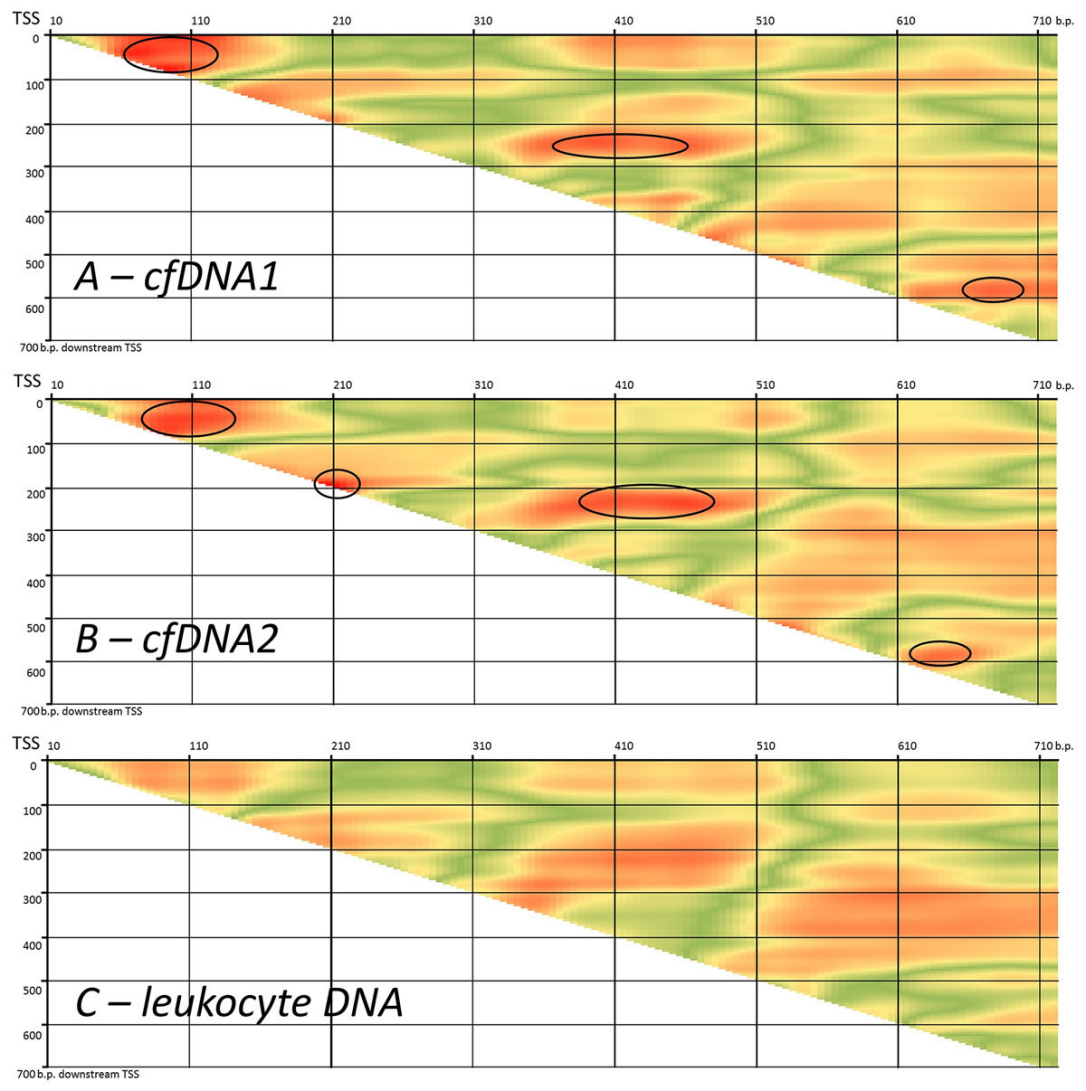
**Overview of statisticaly significant areas in the TSS downstream region**. Pictures *A*, *B *and *C *representes the results for the cfDNA sample 1, cfDNA sample 2 and first patient's leukocyte DNA respectively. Black circles points out combinations of positions which give statistically significant (p-value < 0.05) separation of tissue specific and widely expressed genes.

Previous MNase-seq studies in different human cell lines have shown that there is a correlation between the nucleosome free region, +1 peak coverage and the level of gene expression [28]. To characterize the regions with significant difference in coverage between the cfDNA and genomic DNA, the ratios of the coverage in two different positions were taken for all possible position variations for the 134 tissue specific and 246 housekeeping genes. The positions were selected with a step of 10 base pairs. The null hypothesis was that the sets of ratios that corespond to tissue specific and housekeeping genes could not be differentiated. For each combination of two positions, t-statistics were calculated under the null hypothesis and recorded in the table with numerator coverages in columns and denominator coverages in rows. To visualise the patterns, color coding was employed (Figure 7). Positions that significantly differentiate tissue specific and houskeeping gene datasets are highlighted by black circles (p-value < 0.05). As one can see, no areas of the significant difference were highlighted when gene sets were analyzed in sonicated genomic DNA, while the analysis of the two cfDNA samples resulted in identification of three areas in cfDNA1 and four areas in cfDNA2.

Each area which gives significant differentiation between tissue specific and housekeeping genes (highlighted by black circles in Figure 7) can be associated with the respective nucleosome position and peak in coverage function. The first area was selected for the indepth investigation, as one giving the highest significance rate with average p-value across two cfDNA samples of 0.005 compared witih 0.015 for the second and 0.024 for the third areas respectively. In order to create the function featuring nucleosome fragmentation pattern (which will be able to separate silenced genes from actively expressed) in cfDNA based on coverage function, first peak (after TSS) resolution score was implemented. To calculate it we employed Wolfram Mathematica 9.0 to apply low-pass filter with angular frequency of 0.07 and take the ratio of the resulting coverage of the first peak to the coverage of the subsequent minimum. Peak detection was conducted employing sliding window of 50 b.p. reporting peak if coverage on the edges of window is lower than in the middle. To exclude insignificant fluctuations and noise low pass filter with angular frequency of 0.015 was used before peak detection. If peak or subsequent minimum is not found in restricted window (up to 500 bases downstream TSS) resolution score equals to 1.

**Figure 8:**
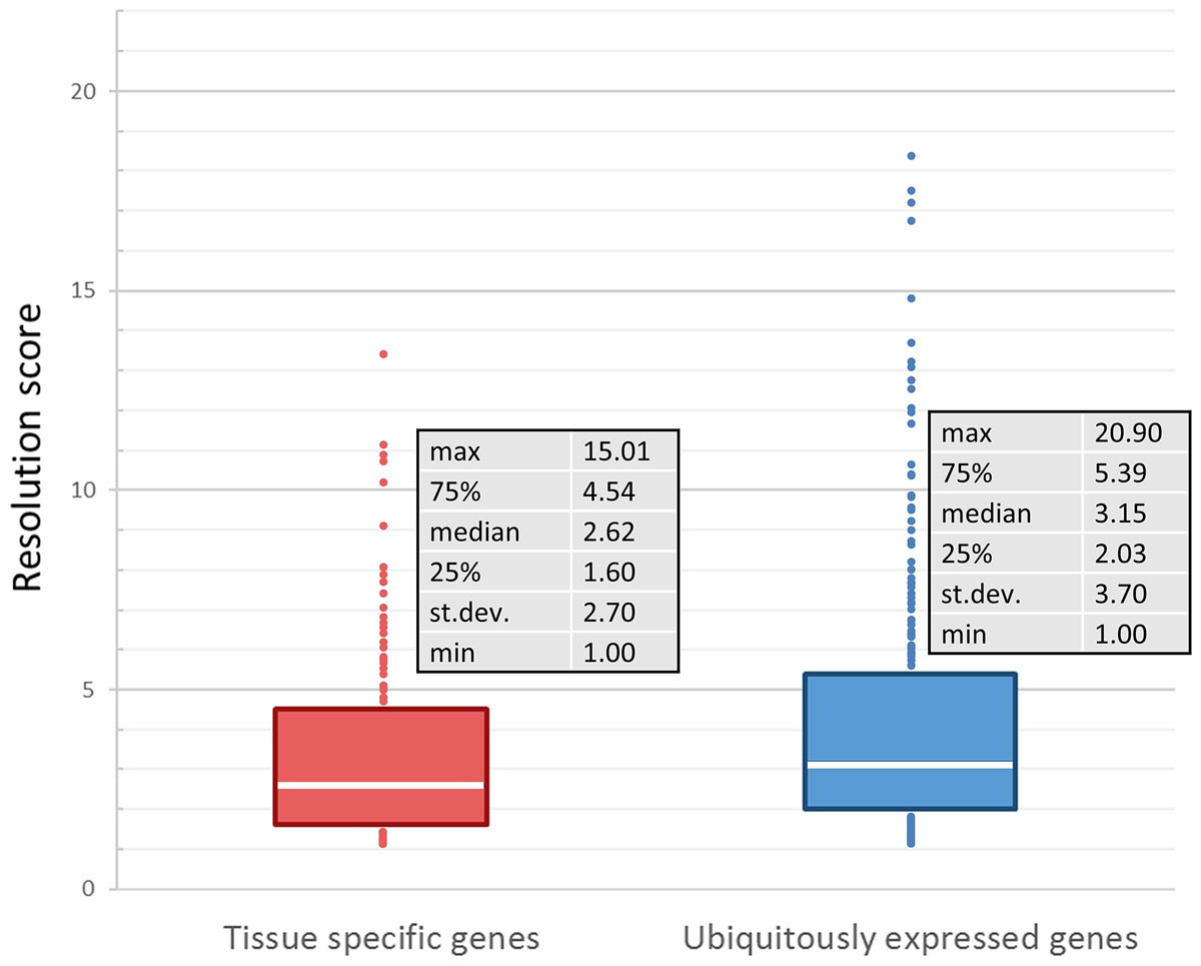
**Resolution score distributions for 134 tissue specific and 246 tissue non-specific genes**. Resolution score is the ratio of the first peak on the coverage function to the subsequent minimum (with the low-pass filter applied).

Further, the resolution score was calculated for the 134 tissue specific genes and 246 household genes among the selected 870 genes and appeared to be good marker which separate tissue-specific from housekeeping genes (Figure 8). For the tissue-specific genes the average resolution score is 3.5, whilst for the ubiquitiously genes - 4.4 (p = 0.007). The same results were obtained for the second patient - significant separation in cfDNA data. If we look at the sonicated genomic DNA, resolution score distributions for tissue specific and household genes do not differ significantly. This indicates, that the first area in Figure 7 is associated with the first peak in coverage function.

Though the model of tissue-specific and household genes as silenced and actively transcribed ones may be rough, statistically significant separation of these two set of genes based on resolution score in cfDNA (whilst no significant result for genomic DNA) indicates that nucleosome fragmentation pattern is associated with gene regulation and measuring the resolution, primary designed to reflect the features of nucleosome fragmentation, we can make judgements about the gene expression status. This makes the cfDNA fragmentation pattern a promising source of biomarkers and further studies should examine the hallmarks of gene expression regulation in cfDNA fragmentation patterns.

## Conclusion

cfDNA have been actively studied recently as a source of different types of diagnostice, predictive and prognostic biomarkers [29-31]. Numerous previous studies have demonstrated signicant differences between normal and cancer cfDNA, including its length, integrity and concentrations [32-35]. Unfortunately, these characteristics are not yet being exploited for biomarker mining. Cancer-specific mutations are being actively studied in cfDNA, though, unfortunately, the sensitivity of their detection in cfDNA is lower than that in tissue biopsy due to the lower concentration of cancer associated DNA [36,37]. In this study, for the first time, the cfDNA nucleosome fragmentation patterns were analyzed and their potential as a source of novel diagnostic biomakers was demonstrated.

It seems that the cfDNA retains characteristics previously noted in genome-wide analysis of chromatin structure. In particular, the fragment size distribution and the read spacing are similar to that obeserved in MNase-seq assays. Moreover, convincing data indicating an association between particular fragmentation patterns of cfDNA and expression regulation, were collected. Interestingly, in a study of the spacing of dinucleosome fragments, two cfDNA fragment histograms were observed. This feature of cfDNA may be of high interest due to its potential value in various diagnostic applications. It seems that cfDNA patterning reflects a general picture of gene expression. Hence, mapping and mining cfDNA fragment ends may aid in the development of novel biomarkers reflecting pathological changes in chromatin marks. The association of fragment copy number with the expression levels in respective locus may aid in detection of various pathologies, including the presence of different types of neoplasms. It is important to note that measuring the copy number of short nucleotide fragments could be, if necessary, performed by qRT-PCR rather than by more expensive sequencing. It is important to note that measuring the copy number of short nucleotide fragments could be, if necessary, performed by qPCR rather than by more expensive NGS.

Moreover, reproducable waving pattern of cfDNA as well as nuclear DNA with high amplitude drops may be used to fine tune the primer positions to achieve higher amplification yields in PCR detection of point mutations in formalin fixed or otherwise degraded samples.

## Conflict of interest

The authors declare that they have no conflict of interest.

## Declaration

Publication of this article has been funded by Ministry of Science and Education, Russia (Project no. RFMEFI60714X0098).

This article has been published as part of *BMC Genomics *Volume 16 Supplement 13, 2015: Selected articles from the 7th International Young Scientists School "Systems Biology and Bioinformatics" (SBB'2015): Genomics. The full contents of the supplement are available online at http://www.biomedcentral.com/bmcgenomics/supplements/16/S13.
